# Effects of Trust, Self-Confidence, and Feedback on the Use of Decision Automation

**DOI:** 10.3389/fpsyg.2019.00519

**Published:** 2019-03-12

**Authors:** Rebecca Wiczorek, Joachim Meyer

**Affiliations:** ^1^Department of Psychology and Ergonomics, Technische Universität Berlin, Berlin, Germany; ^2^Department of Industrial Engineering, Tel Aviv University, Tel Aviv, Israel

**Keywords:** decision aid, alarm system, trust, confidence, feedback, function allocation

## Abstract

Operators often fail to rely sufficiently on alarm systems. This results in a joint human-machine (JHM) sensitivity below the one of the alarm system. The ‘confidence vs. trust hypothesis’ assumes the use of the system depends on the weighting of both values. In case of higher confidence, the task is performed manually, if trust is higher, the user relies on the system. Thus, insufficient reliance may be due to operators’ overconfidence in their own abilities and/or insufficient trust in the decision automation, but could be mitigated by providing feedback. That was investigated within a signal detection task, supported by a system with either higher sensitivity (HSS) or lower sensitivity (LSS) than the human, while being provided with feedback or not. We expected disuse of the LSS and insufficiently reliance on the HSS, in the condition without feedback. The feedback was expected to increase reliance on the HSS through an increase in trust and/or decreases in confidence, and thus, improve performance. Hypotheses were partly supported. Confidence in manual performance was similar to trust in the HSS even though humans’ sensitivity was significantly lower than systems’ sensitivity. While confidence had not effect on reliance or JHM sensitivity, trust was found to be positively related with both. We found disuse of the HSS, that could be improved through feedback, increasing also trust and JHM sensitivity. However, contrary to ‘confidence vs. trust’ expectations, participants were also found to make use of the LSS. This misuse could not be reduced by feedback. Results indicate the use of feedback being beneficial for the overall performance (with HSS only). Findings do not support the idea that misuse or disuse of the system may result from comparison of confidence and trust. We suppose it may rather be the product of users’ wrong strategy of function allocation, based on the underlying idea of team work in combination with missing assignment of responsibility. We discuss this alternative explanation.

## Introduction

In many safety-related work environments, such as process industries, aviation, or health care, operators have to continuously monitor the situation and the underlying processes. They are usually supported by alarm systems which warn them of critical events or deviations from the normal operating state.

Although there are very few accidents in *high reliability organizations* (e.g., Roberts and Rousseau, 1989; Desai et al., 2016; Schulmann, 2016; Martínez-Córcoles et al., 2018), the consequences of accidents in such environments can be severe. Unfortunately, accidents in these highly technologically driven fields are often the result of inadequate responses to available alarms (e.g., Bransby and Jenkinson, 1998; Bliss, 2003; NTSB, 2006).

One common problem is the lack of reliance on the alarm systems, which decreases performance (e.g., Bartlett and McCarley, 2017). The current study aims to investigate whether providing performance feedback represents an adequate countermeasure to this problem. While some studies already used feedback (e.g., Onnasch et al., 2014a), it has never been systematically investigated, whether feedback really improves reliance and thus performance. Furthermore, we want to understand the effect of feedback on the underlying concepts of self-confidence and trust.

### The False Alarm Problem

One of the potential reasons for the disuse (i.e., insufficient use of automation) of alarm systems is the frequency of *false alarms* (Parasuraman and Riley, 1997). The reason for the large number of false alarms in almost all safety-related work environments is the *fail-safe approach*, which aims to minimize the number of critical events that are missed (Swets, 1992). Alarm thresholds are usually set very low, and systems generate alarms whenever they detect the slightest deviation from the normal state. But in most cases these alarms then turn out to be false. The frequent experience of false alarms can decrease the operators’ trust in alarm systems (e.g., Lee and See, 2004; Madhavan et al., 2006; Rice, 2009). This can lead to the disuse of the system in terms of longer reaction times and a decrease in the tendency to respond to alarms (e.g., Bliss et al., 1995; Getty et al., 1995; Dixon et al., 2007; Chancey et al., 2017) – what has been referred to as the *cry wolf phenomenon* (Breznitz, 1984). Also the opposite effect can be observed. In case of high trust, operators rely more on the automation (e.g., Lees and Lee, 2007; Körber et al., 2018).

### Disuse Through Miscalibration of Information

Ideally, users should use alarm systems so that the joint human machine (JHM) performance is better than the performance of either the human or the alarm system alone (Sorkin and Woods, 1985). They should perform the task manually if their sensitivity (discrimination ability) is higher than the system’s, because the use of unreliable automation results in decreased performance (Chavaillaz and Sauer, 2017). However, if the systems’ sensitivity exceeds the users’, they should rely on the system, even though it is not perfectly reliable. Accordingly, some researchers have suggested a theoretical framework based on the ‘confidence vs. trust hypothesis.’ That is, the use of the alarm system may depend on the comparison of trust in the alarm system with operators’ confidence regarding their own abilities. Whenever trust in the system exceeds self-confidence, operators would rely on the system, whereas when the level of trust was lower than the level of self-confidence, they would base their decisions on their own interpretation of the available information (Lee and Moray, 1992; Madhavan and Wiegmann, 2007).

However, most studies find a tendency to disuse automation. That is, participants perform the task manually to a greater extent than warranted and do not sufficiently use the advices of the alarm system. As a consequence, the JHM performance has often been found to be suboptimal in that the overall sensitivity was lower than the sensitivity of the system alone (e.g., Dixon and Wickens, 2006; Rice et al., 2010; Meyer et al., 2014; Bailey, 2015; Bartlett and McCarley, 2017). When participants notice that an alarm system is not perfectly reliable, they often tend to reduce compliance (Meyer, 2004) with alarms and rely on their own ability to discriminate between critical and normal events. This might be the result of miscalibration, either based on insufficient trust or on overconfidence. The latter is a well-known bias in decision-making research.

### Overconfidence Bias in Behavioral Decision-Making

A large body of literature on behavioral decision-making deals with people’s ability to judge the quality of their own decision-making. One consistent finding over the years has been the appearance of overconfidence, defined as a tendency to be more confident in the correctness of one’s own decisions than is actually warranted (e.g., Yates, 1990; Harvey, 1997; Klayman et al., 1999). Overconfidence represents a problem in many domains and was investigated especially in economic and political decision making (Ortoleva and Snowberg, 2015; Ringuest and Graves, 2017; Tsai et al., 2018). One aspect of such overconfidence is the tendency to overestimate one’s own performance in a task (Moore and Healy, 2008; Merkle and Weber, 2011), which is more pronounced for difficult tasks than for easy ones (Larrick et al., 2007). In addition, it has been found that if participants receive more information relevant to the task, their overconfidence increases to a greater extent than their accuracy (Tsai et al., 2008). However, there are differences both in the degree of overestimation for different tasks, as well as between individuals (Klayman et al., 1999).

### Feedback as a Countermeasure for Reducing Overconfidence

Some researchers have shown that providing performance feedback is an operant way to reduce overconfidence (e.g., Arkes et al., 1987; O’Connor, 1989; van Loon and Roebers, 2017), whereas others did not find this positive effect of feedback (e.g., Subbotin, 1996; Pulford and Colman, 1997; Zamary et al., 2016). Stone and Opel (2000) found different effects of various types of feedback. While a treatment that was referred to as environmental feedback, representing a sort of training with task-relevant information, led to an increase in overconfidence, performance feedback on the actual decision-making could reduce the overconfidence. Additionally, it was shown that feedback about the reliability of the task-relevant information could help to reduce overconfidence of the decision-maker (Bregu, 2017).

In alarm research, the effect of feedback on use and performance has not yet been systematically investigated. Nevertheless, different studies employed various ways to provide feedback. The most common approach is to provide feedback after each trial. This can either be done by directly notifying the users about whether their decision was correct or not, or by informing them if the indication given by the alarm system was right or wrong (e.g., Bliss et al., 1995; Getty et al., 1995; Madhavan et al., 2006; Dixon et al., 2007; McBride et al., 2011; Rice and McCarley, 2011; Meyer et al., 2014). Some paradigms provide indirect feedback, where the appropriateness of the alarm and the participant’s decision can be inferred from cross-checking underlying information (i.e., signal present or absent) or by monitoring the further development of the situation (e.g., Bustamante et al., 2007; Bahner et al., 2008; Möller et al., 2011; Onnasch et al., 2014a). A few studies did not give instant feedback after each decision, but provided summarized feedback at the end (e.g., Manzey et al., 2014; Wiczorek and Manzey, 2014), which represents the condition in many real-world scenarios.

It is reasonable that feedback could improve system use, either through the increase of trust in the automation, or through the decrease of (over-)confidence, or even both. We consider it important to understand these underlying mechanisms as base for developing efficient ways of providing appropriate feedback to users of alarm systems.

### The Experiment

In the current experiment we aim to investigate the impact of feedback when working with automation of different sensitivity on the use of automation and resulting performance. Furthermore, we want to understand whether these potential effects result from changes in trust, confidence or both.

Two alarm systems with different sensitivity were used by the participants in the current experiment and outcomes were compared with the manual performance of the participants. The systems are designed in a way that the sensitivity of the one (high sensitivity system, HSS) exceeds the humans’ sensitivity, while the sensitivity of the other system (low sensitivity system, LSS) remains below. While the two alarm systems will be modeled based on signal detection theory (SDT, Green and Swets, 1966), the mean sensitivity of the human subjects can be achieved through adaptation of stimulus material. The two conditions (HSS vs. LSS) will be further divided in one group receiving feedback and another group without. After training the task alone, a manual performance block will be conducted, followed by a system performance block with prior training with the system. Sensitivity and reliance serve as objective measures, complemented by subjective assessment of confidence and trust. While confidence is usually measured through participants’ estimated performance, trust is often assessed on a multidimensional questionnaire. In order to compare both measures with each other, we decided to assess both in the same way using comparable single item scales. Hypotheses are:

H1: Following the ‘confidence vs. trust’ hypothesis, higher confidence should reduce reliance on the system, while higher trust should increase reliance. Thus, reliance should be positively correlated with trust and negatively correlated with confidence. H2: This should result in higher reliance on the HSS compared to the LSS, which is expected to lead to manual performance. H3: As a result, performance with the system should be higher compared to manual performance when using the HSS and there should not be a difference between manual and system supported performance with the LSS. Accordingly, trust should be positively correlated with performance, while confidence should not. H5: However, in line with prior studies (e.g., Dixon and Wickens, 2006; Rice et al., 2010; Meyer et al., 2014), we expect a certain disuse in terms of insufficient reliance on the HSS, indicating a general tendency for overconfidence, that is known from decision making research (e.g., Yates, 1990; Harvey, 1997; Klayman et al., 1999), and/or insufficient trust in the alarm system. H6: Providing feedback should improve participants’ use decisions. They should keep or even reduce the low reliance on the LSS, while increasing reliance on the HSS. H7: This effect should be achieved through an increase of trust in the system, or a decrease of (over-)confidence, or both. H8: Changes in reliance should rise performance. Thus, feedback should lead to a better performance in terms of JHM sensitivity.

## Materials and Methods

“Tel Aviv University Ethics Committee for Research with Human Subjects” approved the study under the name: “Decision making with alerting systems” (no serial numbers available). All procedures were performed in accordance with the Declaration of Helsinki, in compliance with relevant laws, institutional guidelines. Informed consent was obtained from each participant and privacy rights were observed.

### Participants

Eighty students from Tel Aviv University participated in the experiment. We recruited the participants through ads that were put out on campus and through student electronic bulletin boards. We paid them a show-up fee for participating in the experiment of 40 ILS (US $10). Additionally, participants took part in a lottery of four times 100 ILS (about $28.00). Each point in the performance score was a lottery ticket for this lottery, so that participants had an incentive to collect as many points as possible. Their background was in mechanical engineering, industrial engineering and management, political science, and biomedical engineering. Their ages ranged from 19 to 32 years (*M* = 24.25, *SD* = 2.25), 43 were male and 37 female. They were randomly assigned to one of four experimental groups of 20 participants each. They were informed about the purpose of the study without receiving too much detailed information that would affect the results.

### Design

The experiment consisted of a 2 (Sensitivity) × 2 (Feedback) between-subjects design. Half of the participants worked with the LSS and the other half interacted with a HSS. Half of each sensitivity group received visual feedback after each decision during the experimental blocks, while the other half received no feedback. All participants first conducted the task manually, as a baseline, half of them with feedback, the other half without, and then performed with the support of an alarm system. Before, participants of all groups were trained manually and with the system where all of them received feedback to get familiar with the task and the automation.

### Task

**FIGURE 1:**
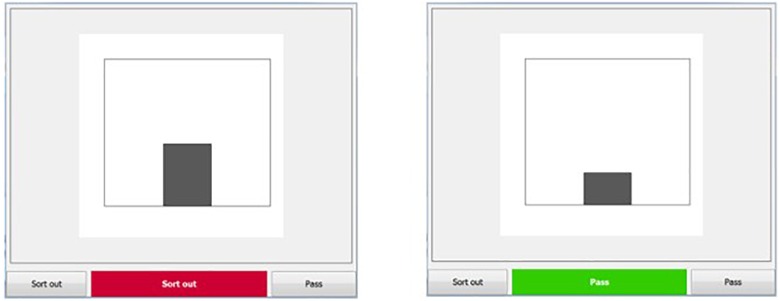
Simulation environment.

The simulation environment has been used before (Meyer et al., 2014). Participants carried out a signal detection task on a 22″ Screen. The task was introduced as a quality control task, similar to those in manufacturing. Participants saw pictures containing vertical bars until they responded or up to 20 s. Then the simulation proceeded to the next picture. The lengths of the bars in the pictures were drawn from two normal distributions with different mean lengths. One distribution represented the intact products and the other the faulty ones. The task was to decide whether to sort out the product or to let it pass based on its length. Participants had to indicate their decision by clicking the corresponding button below the picture (see Figure 1).

In the system performance blocks, an automated decision aid supported participants by showing a cue under the picture 1 s after the picture onset. The cue was either a red or a green horizontal bar with the word ‘Sort out’ or ‘Pass,’ respectively. In the feedback conditions, visual feedback was provided after each decision (i.e., ‘you were right’ vs. ‘you were wrong’).

### Alarm Systems

While participants performed the task unaided in the manual performance block, they were supported by one of two alarm systems in the system performance block. Both systems were designed based on SDT. The SDT derives of the field of psycho physics. It describes the detection of signals in noisy environments. Therefore, the noise and the signal (noise+signal) are represented as two overlapping density distributions. SDT allows the differentiation between the sensitivity and the criterion (response bias) of the alarm system (Egan, 1975). The distance between the two curves represents the sensitivity *d’* of the system, while the criterion *c* gives information regarding the systems proclivity toward alarms or their absence. The two decision aids differed with regard to their sensitivity *d’*. Both had a neutral criterion *c*, leading to an equal number of misses and false alarms. That was achieved by keeping the positive predictive value (PPV) and the negative predictive value (NPV) equal. The PPV represents the conditional probability of the presence of a signal, given a red cue (Getty et al., 1995). The NPV indicates the conditional probability of the absence of a signal, given a green cue (Meyer and Bitan, 2002).

The underlying base rate of faulty products was 0.5. The HSS had a PPV and NPV of 0.9, each, deriving from a *d’* of 2.6. The LSS had a PPV and NPV of 0.7 and a corresponding sensitivity of 1.05.

### Creation of Stimuli

We designed the experiment so that participants would encounter either an alarm system with a sensitivity higher than their own or one with a sensitivity lower than their own. The calculated medium PPV/NPV (between 0.9 and 0.7 of the two systems) is 0.8. With the same base rate of 0.5 it results in a set value of *d’* = 1.68. This set value we tried to achieve by manipulating the stimulus material (i.e., changing length of the bars of the two distributions).

The final stimulus material was evaluated in a pretest with five participants. The analysis showed a mean *d’* of *M* = 1.65 with a standard deviation *SD* = 0.15. One sample *t*-tests (two-tailed), with the significance level set to *p* = 0.2 for null hypothesis testing, indicated no difference between the calculated *d’* = 1.68 and the observed value, *t*(4) = −0.49, *p* = 0.648. Thus, it was decided to use those pictures for the experiment.

### Payoff

For every correct decision (i.e., letting an intact product pass and sorting out a faulty product) participants gained ten points. They lost ten points for every wrong decision (sorting out intact products or letting faulty ones pass). Participants could join a lottery after the experiment, where their score determined the probability of winning a small cash prize.

### Measures

• *Confidence ratings* were assessed twice with a single item questionnaire after the manual performance block and again after the system performance block. Participants were asked how confident they were regarding their decisions during the previous block. Answers were assessed on a 5-point Likert-scale, ranging from ‘not confident at all’ to ‘very confident.’
• *Trust ratings* were assessed twice with a single item questionnaire after the system training block and again after the performance block. Participants were asked how much they trusted the system they had worked with. Answers were assessed on a 5-point Likert-scale, ranging from ‘not at all’ to ‘very much.’
• We recorded participants’ decisions, and used these to calculate behavior measures, based on SDT. *Reliance and compliance* are the two different responses toward the binary cues of an alarm system. According to Meyer (2004) compliance refers to following the alarm system in case it generates an alarm (e.g., red cue) and engage in action, while reliance means refraining from any action in the absence of an alarm (e.g., green cue). Compliance and reliance were calculated using the manual performance block (i.e., baseline) as reference, as suggested by Meyer et al. (2014). The difference between participants’ internal cut-off setting (i.e., criterion c) in the manual performance block (c_manual_) and their cut-off settings in the system performance block with green and red cues (c_system/green_ and c_system/red_) constitutes reliance and compliance, respectively. We calculated c using the z-transformations of the hit-rate, which is based on the proportion of hits out of all signal events, and the false alarm-rate, which is based on the proportion of false alarms out of all noise events:
  (1) $$\mathrm{pHit}=\frac{\mathrm{hits}}{\mathrm{hits}+\mathrm{misses}}$$
  (2) $$\mathrm{pFA}=\frac{\mathrm{FAs}}{\mathrm{FAs}+\mathrm{CRs}}$$
  (3) c=−0.5 (z[pFA]+z[pHit])
  (4) $$\mathrm{compliance}=c_{\text{manual}}-c_{\text{system}/\text{red}}$$
  (5) $$\mathrm{reliance}=c_{\text{system}/\text{green}}-c_{\text{manual}}$$

Reliance is observed when participants’ cut-off with green cues (i.e., indicating the decision to let the product pass) is higher (i.e., more conservative) than their cut-off without any cues. Compliance means the liberalization (i.e., lowering) of cut-offs with red cues. Both have positive values, with higher values indicating stronger compliance or reliance, pointing to a greater tendency to follow the system’s advice.

• *Sensitivity d’* was calculated (based on participants’ decisions using SDT) by subtracting the z-transformed hit-rate from the z-transformed FA-rate:
  (6) d'=z(pHit)−z(pFA)

Sensitivity in the manual performance block describes the human’s discrimination ability, while JHM *d’* in the system performance block represents the joint human-machine sensitivity.

### Procedure

**FIGURE 2:**
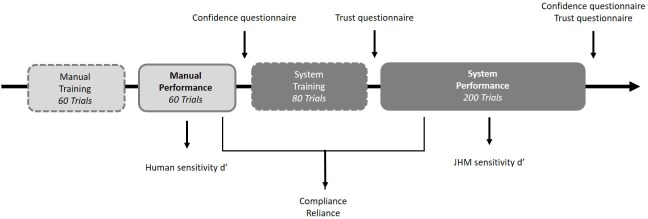
Procedure of the training and experimental blocks, and dependent measures.

The whole experiment lasted about 1 h. Participants first signed a consent form and received standardized instructions on a PC screen. The experiment consisted of a manual phase (training block and performance block) and a system phase (training block and performance block). Participants initially performed a manual training (60 trials). During the training phase all of them received online feedback after each decision to become familiar with the task. They then performed the manual experimental block consisting of 60 trials, with only half of the participants receiving online feedback. Participants then completed the confidence questionnaire. They were then trained with the system they would use afterward. The system training phase consisted of 80 trials, and all participants received online feedback after each decision to become familiar with the system. After the training they completed the trust questionnaire. They then performed the system experimental block with 200 trials. Only half of the participants received online feedback. After the system experimental block, participants completed the confidence questionnaire and the trust questionnaire again. At the end of the experiment, participants were thanked and dismissed. They were also given the possibility to ask questions about the study. An overview of the procedure is displayed in Figure 2.

## Results

We used an alpha level of 0.05 for all statistical tests unless otherwise stated. To analyze data from the system performance block, we conducted ANOVAs with *Sensitivity* and *Feedback* as between-subject factors, and with the *Behavioral tendency*, compliance and reliance, as a within subject factor. We used *t*-tests for independent samples (two-tailed) to analyze the data from the manual performance block to compare the groups with and without feedback, and for the trust assessments after the system training phase to compare the groups with the HSS and the LSS. *T*-tests were also used for *post hoc* comparisons, which served to further investigate interaction effects and to compare manual and system performance. Means and standard deviations of all dependent measures can be found in Table 1.

**Table 1:** Means (and standard deviations in brackets) of all dependent measures for the manual performance block and system performance block (separate for HSS and LSS) with and without feedback.

	Manual performance block *(system training block)*		System performance block			
	With feedback	Without feedback	HSS		LSS	
			With feedback	Without feedback	With feedback	Without feedback
Confidence	3.5 (0.8)	3.4 (0.7)	4 (0.8)	3.2 (0.5)	3.3 (0.7)	3 (0.8)
Trust	*3.4 (0.7)*	*2.5 (0.6)*	3.4 (1.0)	3.3 (0.9)	2.1 (0.7)	2.4 (0.5)
Compliance	–	–	1.1 (0.6)	0.8 (0.6)	0.6 (0.5)	0.5 (0.4)
Reliance	–	–	1.2 (0.6)	0.6 (0.6)	0.5 (0.3)	0.2 (0.3)
Sensitivity *d’*	1.8 (0.4)	1.8 (0.3)	2.5 (0.3)	2 (0.7)	1.6 (0.2)	1.6 (0.3)

*Trust ratings in italics derive from the system training block.*

### Confidence Ratings

#### Manual Performance Block

The analysis did not reveal a significant difference between the groups with feedback (*M* = 3.45) and without feedback (*M* = 3.40). The confidence in decisions did not differ as a function of feedback.

#### System Performance Block

Analyses revealed significant main effects of *Sensitivity F*(1,76) = 6.8, *p* = 0.011, $\eta_{p}^{2}$ = 0.08, and of *Feedback*, *F*(1,76) = 10.38, *p* = 0.002, $\eta_{p}^{2}$ = 0.12. The interaction effect did not reach significance.

**FIGURE 3:**
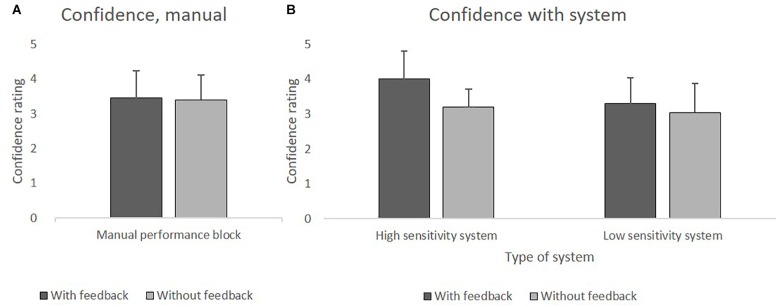
**(A)** Mean values of confidence in the manual performance block with and without feedback. **(B)**. Mean values of confidence using HSS and LSS with and without feedback.

Participants’ confidence was higher when receiving feedback. That was true for the HSS (with feedback *M* = 4 vs. without feedback *M* = 3.2), as well as for the LSS (with feedback *M* = 3.3 vs. without feedback *M* = 3). On average, HSS led greater confidence than did LSS. Figure 3A,B depict the mean values of confidence.

Additionally, we used one sample *t*-tests (two-tailed) to compare the confidence of the system performance block with the mean confidence of the manual performance block (*M* = 3.48). The Bonferroni-corrected significance level of *p* = 0.0125 was applied. When using the HSS with feedback, confidence was significantly higher than when performing the task manually *t*(19) = 3.21, *p* = 0.005. Confidence in the other three conditions of the system performance block did not differ significantly from the manual performance block.

### Trust Ratings

#### System Training Phase

**FIGURE 4:**
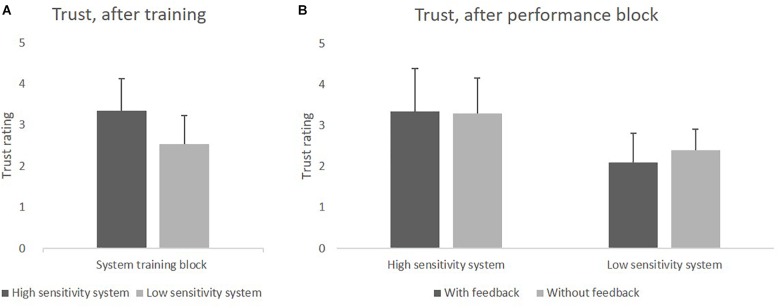
**(A)** Mean values of trust in the HSS and LSS after the training block. **(B)** Mean values of trust after using HSS and LSS with and without feedback in the system performance block.

Trust in the system, assessed after the training phase, was analyzed with a *t*-test for independent samples (two-tailed), comparing the two groups with HSS and LSS. Participants who were trained with the HSS had more trust in the system (*M* = 3.35) than those who were trained with the LSS (*M* = 2.53), *t*(78) = 5.85, *p* < 0.001. Results can be seen in Figure 4A.

#### System Performance Block

The main effect of *Sensitivity* was found to be significant, *F*(1,76) = 35.59, *p* < 0.001, $\eta_{p}^{2}\eta_{p}^{2}$ = 0.32, while the main effect of *Feedback* and the interaction effect were not. Trust in the system was higher for the HSS (*M* = 3.33) than for the LSS (*M* = 2.25), and feedback had no impact on trust. Results can be seen in Figure 4B.

Additionally, we used one sample *t*-tests (two-tailed) to compare trust assessed in the system training block with trust assessed in the system performance block for trust in the HSS and trust in LSS, separately. The Bonferroni-corrected significance level of *p* = 0.025 was applied. Trust in HSS did not change between training and performance block, whereas trust in LSS decreased significantly, *t*(39) = −2.81, *p* = 0.009.

### Comparison of Confidence and Trust

We used one sample *t*-tests (two-tailed) to compare the trust ratings after the training phase with the mean confidence of the manual performance block (*M* = 3.48) to investigate the ‘confidence vs. trust hypothesis.’ The Bonferroni-corrected significance level of *p* = 0.025 was applied. It was found that trust in the HSS did not differ significantly from confidence in the manual performance block, while trust in the LSS was significantly lower, *t*(39) = −10.9, *p* < 0.001.

### Reliance and Compliance

#### System Performance Block

**FIGURE 5:**
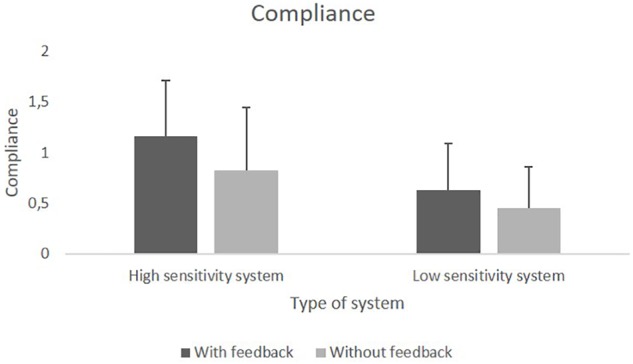
Mean values of compliance with the HSS and the LSS with and without feedback.

We found a significant main effect of *Sensitivity*, *F*(1,76) = 34.95, *p* < 0.001, $\eta_{p}^{2}\eta_{p}^{2}$ = 0.32, as well as a significant main effect of *Feedback, F*(1,76) = 16.46, *p* < 0.001, $\eta_{p}^{2}\eta_{p}^{2}$ = 0.18. Neither the main effect of *Behavioral Tendency*, nor any of the interaction effects reached significance. Both compliance and reliance were higher for the HSS compared to the LSS. Feedback increased both compliance and reliance compared to no feedback. Figure 5, 6 show the means for compliance and reliance.

**FIGURE 6:**
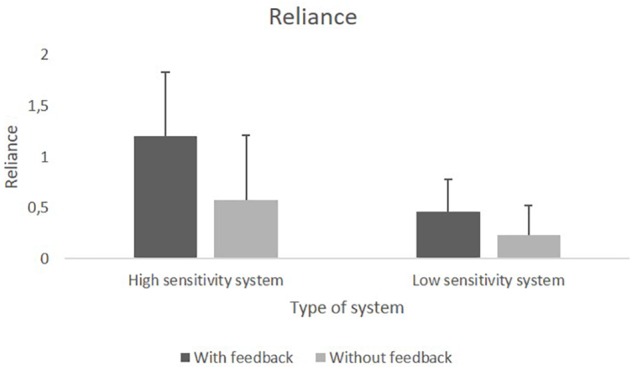
Mean values of reliance on the HSS and the LSS with and without feedback.

### Sensitivity (d’)

#### Manual Performance Block

The human sensitivity *d’* did not differ significantly between the group with feedback (*M* = 1.78) and the group without feedback (*M* = 1.79). However, additionally one sample *t*-tests (two-tailed) indicated that participants’ sensitivity *d’* was significantly lower than the one of the HSS, *t*(79) = −18.97, *p* < 0.001, as well as significantly higher than the one of the LSS, *t*(79) = 17.79, *p* < 0.001.

#### System Performance Block

Analyses of the JHM *d’* revealed significance for the main effect of *Sensitivity, F*(1,76) = 46.32, *p* < 0.001, $\eta_{p}^{2}$ = 0.38, for the main effect of *Feedback*, *F*(1,76) = 6.81, *p* = 0.01, $\eta_{p}^{2}$ = 0.08, and for the interaction effect of *Sensitivity* × *Feedback*, *F*(1,76) = 6.26, *p* = 0.02, $\eta_{p}^{2}\eta_{p}^{2}$ = 0.08. *Post hoc* single comparisons were made to further investigate the interaction effect by comparing the two *Feedback* conditions (with and without feedback) separately within each system sensitivity group. The Bonferroni-corrected significance level of *p* = 0.025 was applied. In the HSS group a significant difference was found for the conditions with feedback (*M* = 2.48) and without feedback (*M* = 2), *t*(38) = 2.75, *p* = 0.009, whereas in the LSS group the condition with feedback (*M* = 1.61) and without feedback (*M* = 1.60) did not differ significantly.

**FIGURE 7:**
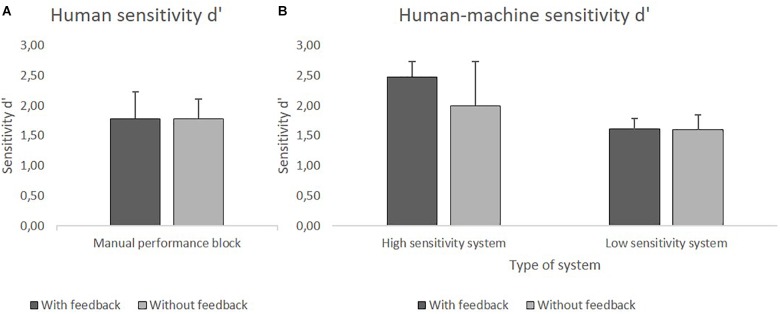
**(A)** Mean values of human sensitivity *d’* in the manual performance block with and without feedback. **(B)** Mean values of JHM sensitivity *d’* using HSS and LSS with and without feedback.

The JHM sensitivity *d’* was higher when participants used the HSS compared to the LSS. Availability of feedback led to a higher performance, but that was true only for the HSS group. Figure 7A,B show the mean *d’* values.

Additionally, one sample *t*-tests (two-tailed) were conducted to compare the different JHM sensitivities with the mean human sensitivity of the manual performance block (*M* = 1.79). The Bonferroni-corrected significance level of *p* = 0.0125 was applied. For the HSS, analyses revealed a significantly higher *d’* with feedback, *t*(20) = 11.87, *p* < 0.001, and no significant difference without feedback. For the LSS, *d’* values were significantly lower both with feedback, *t*(20) = −4.53, *p* < 0.001, and without feedback, *t*(20) = −3.39, *p* = 0.003.

Only the combination of a HSS and performance feedback could improve *d’* over manual performance. In contrast, the use of the LSS did worsen performance.

### Correlation of Trust and Confidence With Behavior and Performance

Trust assessed in the training block was found to be positively correlated with reliance, *r* = 0.31, *p* = 0.005, but not compliance in the system performance block, and also positively correlated with JHM *d’* in the system performance block, *r* = 0.32, *p* = 0.004. That means the higher the prior trust in the system the more participants rely on the green cues (i.e., ‘Let pass’), which rise JHM *d’*. In contrast, no correlation between confidence in the manual performance block and compliance, reliance or *d’* in the system performance block was found. Thus, trust can serve as predictor of behavior and performance while confidence does not.

## Discussion

The purpose of the study was to investigate, whether performance feedback has the potential to mitigate the disuse of alarm systems, and to understand how the feedback affects the underlying components of self-confidence and trust. We compared participants’ use of assistance systems with high and low sensitivity with and without feedback with regard to system use and resulting performance, while assessing confidence in participants’ decisions as well as trust in the system.

### Behavior and Performance

When no feedback was available, participants made some use of the HSS, as measures of compliance and reliance show, but not in a sufficient way. JHM sensitivity did not significantly differ from human sensitivity in the manual performance block, and therefore remained much below the system’s sensitivity. This finding corresponds to our hypothesis that participants would not use the system sufficiently and is in line with prior findings (Dixon and Wickens, 2006; Rice et al., 2010; Meyer et al., 2014; Bailey, 2015; Bartlett and McCarley, 2017). Providing online feedback led to significantly higher values of compliance and reliance with the HSS, which significantly increased JHM sensitivity. However, the performance value was still below the sensitivity which could have been achieved when completely relying on the system.

Participants who were supported by the LSS should have done the task manually, ignoring the system as it could not increase their sensitivity. However, they still did rely to some extent on the system. This behavior was detrimental, because the JHM sensitivity was significantly lower than participants’ unaided sensitivity. The availability of feedback had no significant impact on participants’ behavior and the resulting performance when using the LSS.

### Confidence and Trust

Confidence ratings in the manual block did not differ as a function of the availability of feedback. This result is in line with other studies in not finding a confidence reducing effect of feedback (e.g., Subbotin, 1996; Pulford and Colman, 1997). However, the manual performance block was preceded by training with feedback which corresponds to the two types of feedback (environmental and performance) described by Stone and Opel (2000). Thus, it is possible that the training block already led to a reduction of preexisting overconfidence for all the participants.

Trust in the system training block varied according to system sensitivity. A comparison of confidence in the manual performance block vs. trust in the system training block was made to further investigate the ‘trust vs. confidence’ hypothesis (e.g., Lee and Moray, 1992; Madhavan and Wiegmann, 2007). Confidence of participants in their own decisions (*M* = 3.48) did not differ significantly from trust in the HSS (*M* = 3.35) even though system’s *d’* exceeded the humans’ sensitivity (*d’* = 2.6 vs. *d’* = 1.79, respectively). This miscalibration between confidence and trust could either be the result of overconfidence or undertrust. In any case, it was accompanied by the disuse of the system. In contrast, while trust in the LSS was below confidence, participants did not decide to do the task completely manually, but rather they relied on the system to some extent. Thus, the current experiment does not provide evidence for the ‘trust vs. confidence’ hypothesis.

Contrary to prior effects of feedback on confidence (e.g., Arkes et al., 1987; O’Connor, 1989), the feedback did not reduce confidence in the system performance block. Instead, it led to an increase in confidence. However, confidence reported after the system performance block referred to decision-making in a task supported by an alarm system. Thus, it is possibly an integration of participants’ and systems’ abilities rather than confidence in participants’ abilities alone. In future studies it is recommended to further discriminate between contribution of system and contribution of human to the overall confidence.

Unlike their confidence, participants’ trust ratings were unaffected by feedback. Both confidence and trust increased as a function of system sensitivity.

Correlations of trust and confidence with behavior and performance also contradict the ‘confidence vs. trust’ hypothesis. Prior trust was found to have a positive impact on reliance, which is in line with previous studies (e.g., Lees and Lee, 2007; Körber et al., 2018). However, no negative correlations with prior confidence and compliance or reliance where found that could have supported the hypothesis.

### Potential Alternative Reasons for the Non-optimal Use of Decision Aid

In the current study we found both disuse and misuse (i.e., exceeded use) of assistance systems. Thus, we conclude the existence of a general tendency for miscalibration of the weight given to information from a decision aid that can manifest itself in different ways. It can lead to the insufficient use of highly sensitive automation, as well as to the excessive use of LSS. The miscalibration of trust and confidence *may* play a role in this context. However, the current results do not fully support this assumption, and other possible explanations should be considered.

When comparing our results from human-machine interaction with those from research on human-human interaction, we found similar findings in the study of Bahrami et al. (2010). They also showed that the combination of two decision makers with different sensitivities remained below the performance level of the better one. Additionally, they could show that communication between the parties improved performance even without feedback. This could imply that beyond information about system reliability also information regarding the validity of each single system advise would be needed. This information enables the user to decide for each trial, whether it is suitable to follow the systems advise. For this purpose the use of likelihood alarm systems had been suggested. They communicate their own certainty regarding their advise via graded warnings (e.g., Sorkin et al., 1988; Wickens and Colcombe, 2007).

The current results also correspond with findings from Harvey and Fischer (1997), who investigated whether decision-makers that differ in expertise (novice, medium, experts) take advice from others. The advice-givers also differed with regard to their expertise on the same three levels. The authors conclude that three different mechanisms explain the acceptance of advice. The first mechanism described by Harvey and Fischer (1997) is ‘avoidance of complete rejection.’ Even expert decision-makers accepted approximately 20% of the advice given by novices, because it had presumably been given with a positive intention. This might resemble the unexpected use of the LSS by participants in our experiment. Following this assumption, it is possible that people feel the need to use a system that is introduced to them as assistance or decision aid, even if the quality of the system is low.

The second mechanism described by Harvey and Fischer (1997) is a wish to ‘improve judgment.’ They found that decision-makers increased their acceptance of advice when advice-givers were more experienced, corresponding to our participants relying more on the HSS than on the LSS. More interestingly, their participants, similar to ours, did not use the offered support sufficiently, as not even the novice decision-makers took more than 40% of advice. Harvey and Fischer (1997) explain this with overconfidence, which is analogous to our own initial hypothesis.

However, an alternative explanation should be considered. It is possible that when participants fail to rely sufficiently on a HSS, they follow a similar rationale as those showing overreliance. If the task is assigned to the operators and the system is only there to assist, they might feel the need to contribute to the task regardless whether this is beneficial or not to the overall performance. Thus, the assignment of insufficient significance may be the result of participants’ interpretation of teamwork, i.e., interaction. Perhaps this problem arises when function allocation to the human and the automation is not clearly defined. This assumption also matches the third mechanism of ‘shared responsibility.’ Harvey and Fischer (1997) participants took more advice from others when the task was important, i.e., the risk was high. While we did not vary risk in the current study, Wiczorek and Meyer (2016) showed an increase of manual performance in a human-machine task as a function of risk. Both tendencies, though pointing in different directions, again reflect the concept of teamwork.

Thus, we assume that users’ wrong strategy of function allocation is not the result of overconfidence but the product of participants trying to integrate decisions of both agents in the task to interact as a team.

### Implications for Improving Function Allocation

Our results have several potential implications for the allocation of functions to humans and automation in decision tasks. Firstly, operators supported by decision aids may have a tendency to follow at least some of the advice, and they will also try to contribute to the task themselves. This behavior possibly results from their perception of function allocation, which is based on the idea of teamwork. It will in most cases lead to non-optimal performance (by either assigning too much or too little weight to the information from the decision aid).

Secondly, it may be possible to increase or reduce the reliance on automation in a decision-making task by varied responsibility assignments. This could be done for instance by changing, the level of automation (LOA, Sheridan and Verplank, 1978), at which the aid supports the user. A meta-analysis of Onnasch et al. (2014b) could find effects of LOA on different dependent measures such as workload, situation awareness, etc. As tasks in these studies represent monitoring instead of quality control tasks and systems had very high reliabilities, they were rather focusing on misuse resulting from complacency (see Parasuraman and Manzey, 2010) than on disuse of alarm systems (Parasuraman and Riley, 1997). Thus, the effect of LOA on compliance and reliance with decision automation should be invested in future studies.

Thirdly, humans and decision aids should only form a team when their abilities complement each other. That is the case when both agents base their decisions on different parameters or apply different decision algorithms. If they do not do so, the task should be assigned to the agent with the higher sensitivity.

Lastly, when improvement through complementation is possible, but system sensitivity is much above human sensitivity, methods should be provided to use the human’s expertise, while maintaining the benefits from the high system reliability. This could be achieved by informing operators about the relative competence of each partner and training them to identify situations that are difficult for the system and easy for them, or vice versa. An alternative approach would be the use of likelihood alarm systems (e.g., Sorkin et al., 1988). The users can comply with the high likelihood alerts and do the task manually when the system generates alerts with a low likelihood to be correct, as has been shown by Wiczorek and Manzey (2014). In a more advanced version, the system could execute the task automatically when it is certain and only involve the operator in the decision process when it is uncertain. This approach would have the additional benefit of reducing operators’ workload.

### Limitations

One common limitation that a lot of studies dealing with human-machine interaction faces, it the use of student samples. Real operators are highly trained and possess a lot of experience with their automation, unlike the student volunteers. However, given that the reason for this type of research are real world problems, it is likely to assume that basic behavioral mechanisms and attitudes follow similar mechanisms across operators and students.

Another limitation of the current study is the assessment of confidence after the system performance block. As it was a JHM performance, it is not possible to understand to what extend the indicated confidence derives from the participant’s contribution and the system’s contribution. Future studies may investigate alternative ways of assessing these two components separately.

## Conclusion

When interacting with assistance systems, participants tend to underuse HSSs but overuse low sensitivity ones. Feedback was shown to improve use in the case of HSSs, but not with the LSSs. However, we assume there are not too many reasons to provide operators with a system that performs below their own abilities. If this should happen, feedback will not help to improve user decisions.

Results of the current study do not support the ’confidence vs. trust’ hypothesis. As participants’ decisions fit only one part of our hypothesis (i.e., they do not sufficiently use the HSS), and not the other part (i.e., they do not rely on manual performance with the low-sensitivity system, but rather use its indications excessively), we no longer believe overconfidence to be the (main) reason for effects of disuse. Rather, we suggest that the observed under- and overuse is the result of a wrong strategy of function allocation that is based on the idea of teamwork without clear assignment of responsability. This problem may be resolved by providing additional information regarding function allocation (e.g., assigning explicit levels of automation).

## Author Contributions

RW contributed the study planning, preparation data acquisition, supervising data acquisition, statistical analysis, interpretation, and manuscript writing. JM was involved in the study planning, interpretation, and manuscript revision.

## Conflict of Interest Statement

The authors declare that the research was conducted in the absence of any commercial or financial relationships that could be construed as a potential conflict of interest.
